# Novel Application of Magnetic Protein: Convenient One-Step Purification and Immobilization of Proteins

**DOI:** 10.1038/s41598-017-13648-x

**Published:** 2017-10-17

**Authors:** Min Jiang, Lujia Zhang, Fengqing Wang, Jie Zhang, Guosong Liu, Bei Gao, Dongzhi Wei

**Affiliations:** 1 State Key Lab of Bioreactor Engineering, New World Institute of Biotechnology, East China University of Science and Technology, Shanghai, 200237 China; 2 School of Chemistry and Molecular Engineering, East China Normal University, Shanghai, 200062 China; 3 NYU-ECNU Center for Computational Chemistry at NYU Shanghai, Shanghai, 200062 China; 4 School of Physics and Materials Science, East China Normal University, Shanghai, 200062 China

## Abstract

Recently, a magnetic protein was discovered, and a multimeric magnetosensing complex was validated, which may form the basis of magnetoreception. In this study, the magnetic protein was firstly used in biotechnology application, and a novel convenient one-step purification and immobilization method was established. A universal vector and three linker patterns were developed for fusion expression of magnetic protein and target protein. The magnetic protein was absorbed by iron beads, followed by target protein aggregation, purification, and immobilization. GFP, employed as a reporter protein, was successfully purified from cell lysate. Subsequently, three enzymes (lipase, α-L-arabinofuranosidase, pullulanase) with different molecular sizes testified the versatility of this magnetic-based approach. The specific activities of the purified enzymes were distinctly higher than those of the traditionally purified enzymes using affinity chromatography. The lipase immobilized on iron beads presented improved thermostability and enhanced pH tolerance compared to the free enzyme. The immobilized lipase could be easily recovered and reused for maximum utilization. After 20 cycles of reutilization, the magnetically immobilized lipase retained 71% of its initial activity. This investigation may help introduce magnetic protein into biotechnology applications, and the one-step purification and immobilization method may serve to illustrate an economically viable process for industry.

## Introduction

Whether animals can detect Earth’s magnetic field, and how can they orient or migrate by sensing magnetic field, is one of the most controversial topic. Many researchers have attempted to identify the magnetic sensing and search for magnetic receptor^1–3^. Recently, a magnetic protein biocompass was discovered and reported by Xie, *et al*.^4^. This biocompass is a multimeric magnetosensing rod-like protein complex, consisting of a novel magnetic receptor and magnetoreception-related photoreceptor cryptochromes. The protein complex has been demonstrated to be widely distributed among species, and believed to inspire technology innovation across multiple fields.

Protein separation and purification are important processes in biotechnology^5,6^. Traditional methods, such as isoelectric point, sodium dodecyl sulfate, and gel permeation chromatography have been applied for various kinds of proteins. In the case of low-concentration proteins, separation and purification signify challenging tasks^7,8^. Several fusion tags have been developed and the affinity chromatography has provided an efficient way to purify target fusion proteins^9–11^. Whereas, the high-cost of affinity column and time-consuming manipulation restrict the large-scale usability of this technique. Immobilization of proteins, especially enzymes, is routinely performed since it facilitates the recovery and reuse of expensive enzymes and improves cost-effectiveness^12,13^. Although many attempts have been made to exploit and optimize immobilization methods^14–16^, iron oxide nanoparticles with uniquely large surface-to-volume ratio and quantum size effects offer a quick, convenient, and economical method for immobilizing enzymes^17,18^. Nevertheless, prior enzyme purification is necessary, followed by magnetic immobilization, which places a heavy burden on industrial application. Consequently, one-step purification and immobilization has emerged as required. Ni^2+^-functionalized Fe_3_O_4_@polydopamin nanoparticles were developed for his-tagged proteins^19^. Amino-functionalized MCFs were produced and adhered to aldehyde-tagged enzyme^20^. DNA aptamer-linked magnetic beads were obtained and applied to β-glucuronidases^21^. However, appropriate surface modification of iron oxide nanoparticles or mesocellular siliceous foam should be completed and evaluated before purification and immobilization, and the instability of modified nanoparticles restrict their repeated use.

In this study, the magnetic protein was used in biotechnology application for the first time. A universal vector for fusion expression of magnetic protein and target proteins was constructed, and a method using iron beads (no need for modification) for purification and immobilization of fusion proteins was developed, followed by experimental validation with GFP. Furthermore, several enzymes with different molecular sizes were employed to testify the versatility of this method. The detailed characteristics of immobilization enzyme were investigated as well. Our work provides a novel convenient method that can be exploited for numerous other protein purification and immobilization applications.

## Results

### Heterologous expression and purification of clMagR and dMagR

**Figure 1:**
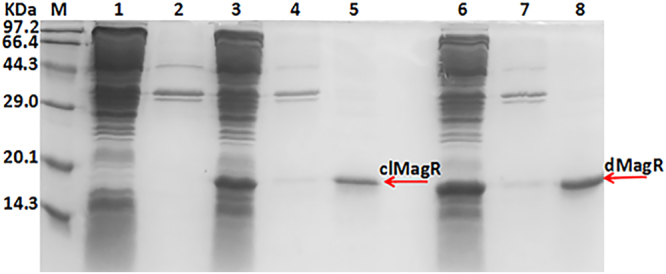
SDS-PAGE analysis of the recombinant MagR and the purified MagR. lane M: standard marker proteins; lane 1: supernatant of cell lysate (BL21-pET28a); lane 2: precipitation of cell lysate (BL21-pET28a); lane 3: supernatant of cell lysate (BL21-pET28a-clMagR); lane 4: precipitation of cell lysate (BL21-pET28a-clMagR); lane 5: the purified clMagR protein; lane 6: supernatant of cell lysate (BL21-pET28a-dMagR); lane 7: precipitation of cell lysate (BL21-pET28a-dMagR). lane 8: the purified dMagR protein.

Recombinant strains (BL21-pET28a-clMagR, BL21-pET28a-dMagR) were grown in LB medium supplemented with appropriate antibiotic. After induction for 18 h, the recombinant clMagR and dMagR were overexpressed as soluble proteins (Fig. 1). Fe_3_O_4_-SiO_2_ nanoparticles were used to absorb and purify the clMagR and dMagR proteins. As shown in Fig. 1, both the purified clMagR and dMagR migrated as a single band on SDS-PAGE with an apparent molecular mass of about 14.6 kDa, which was identical to the calculated value. This result demonstrated the high specificity of the magnetic protein binding to iron beads.

### Linker selection and purification of the fusion protein (clMagR-GFP)

**Figure 2:**
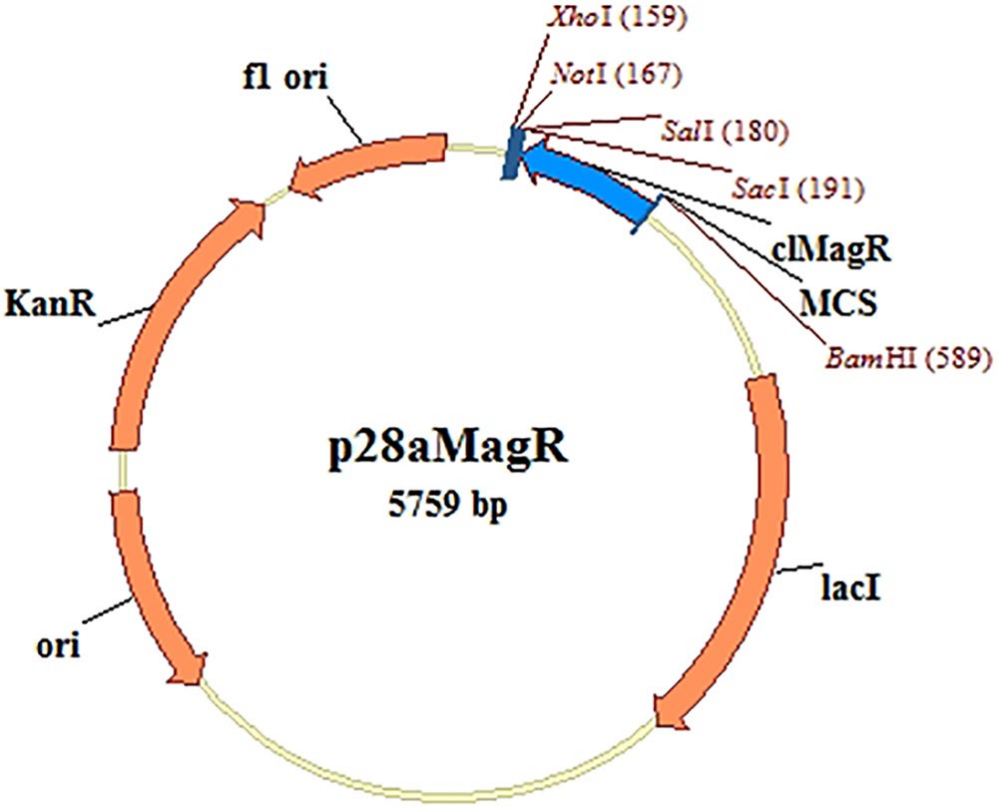
The vector map of p28aMagR.

**Figure 3:**
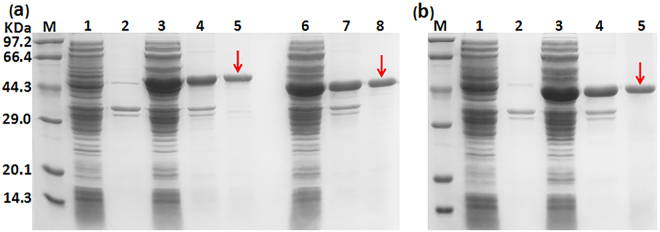
SDS-PAGE analysis of the fusion protein (clMagR-GFP). (**a**) lane M: standard marker proteins; lane 1: supernatant of cell lysate (BL21-pET28a); lane 2: precipitation of cell lysate (BL21-pET28a); lane 3: supernatant of cell lysate (BL21-p28aMagR-rigid linker-GFP); lane 4: precipitation of cell lysate (BL21-p28aMagR-rigid linker-GFP); lane 5: the purified clMagR-rigid linker-GFP protein (the single band marked with the arrow); lane 6: supernatant of cell lysate (BL21-p28aMagR-flexible linker-GFP); lane 7: precipitation of cell lysate (BL21-p28aMagR-flexible linker-GFP); lane 8: the purified clMagR-flexible linker-GFP protein (the single band marked with the arrow); (**b**) lane M: standard marker proteins; lane 1: supernatant of cell lysate (BL21-pET28a); lane 2: precipitation of cell lysate (BL21-pET28a); lane 3: supernatant of cell lysate (BL21-p28aMagR-GFP); lane4: precipitation of cell lysate (BL21-p28aMagR-GFP); lane 5: the purified clMagR-GFP protein (the single band marked with the arrow).

Since clMagR and dMagR exhibited a similar expression level, clMagR was used to construct a universal vector (Fig. 2). In addition, three linker patterns were designed to purify fusion protein. Figure 3 illustrated that clMagR-GFP, clMagR-flexible linker-GFP, clMagR-rigid linker-GFP were all highly expressed in *E*. *coli*, and mainly in soluble forms. After purification with iron beads, the fusion proteins showed an apparent single band of 45 kDa according to SDS-PAGE analysis, which was consistent with the expected molecular mass of clMagR-GFP. Meanwhile, the data from fluorescence microtiter plate reader (Table 1) also indicated that GFP was efficiently expressed and displayed activity. These results indicated that the active GFP was successfully purified from cell lysate through adsorption of clMagR to iron beads.

**Table 1:** Fluorescence intensity of clMagR-rigid linker-GFP, clMagR-flexible linker-GFP and clMagR-GFP.

Samples	BL21 (pET28a)	BL21(p28aMagR -rigid linker-GFP)	BL21 (p28aMaR -GFP)	BL21 (p28aMagR -flexible linker-GFP)
Fluorescence intensity	208 ± 10	48094 ± 1265	42809 ± 1130	42632 ± 1072

Furthermore, as illustrated in Table 1, the fluorescence intensity of clMagR-GFP was similar to that of clMagR- flexible linker-GFP, but only a little lower than that of clMagR-rigid linker-GFP. In view of the simplicity and convenience of manipulation, no linker was added between the magnetic protein and the target protein in the following study.

### The versatility of magnetic protein as purified label

**Figure 4:**
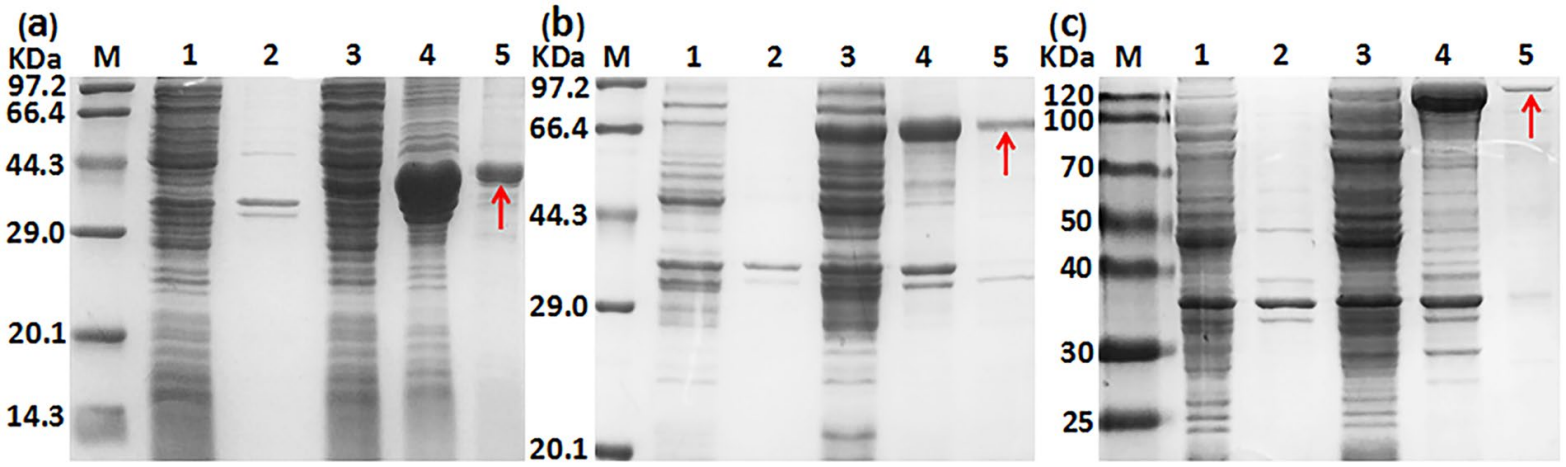
SDS-PAGE analysis of clMagR-lipase, clMagR-α-AF, clMagR-pullulanase. (**a**) lane M: standard marker proteins; lane 1: supernatant of cell lysate (BL21-pET28a); lane 2: precipitation of cell lysate (BL21-pET28a); lane 3: supernatant of cell lysate (BL21-p28aMagR-lipase); lane 4: precipitation of cell lysate (BL21-p28aMagR-lipase); lane 5: the purified clMagR-lipase protein (the single band marked with the arrow); (**b**) lane M: standard marker proteins; lane 1: supernatant of cell lysate (BL21-pET28a); lane 2: precipitation of cell lysate (BL21-pET28a); lane 3: supernatant of cell lysate (BL21-p28aMagR-α-AF); lane 4: precipitation of cell lysate (BL21-p28aMagR-α-AF); lane 5: the purified clMagR-α-AF protein (the single band marked with the arrow); (**c**) lane M: standard marker proteins; lane 1: supernatant of cell lysate (BL21-pET28a); lane 2: precipitation of cell lysate (BL21-pET28a); lane 3: supernatant of cell lysate (BL21-p28aMagR-pullulanase); lane 4: precipitation of cell lysate (BL21-p28aMagR-pullulanase); lane 5: the purified clMagR-pullulanase protein (the single band marked with the arrow).

To expand the application of this purification method, three enzymes with different molecular sizes (lipase gene 549 bp, α-AF gene 1479 bp, pullulanase gene 2766 bp) were chosen as target proteins. SDS-PAGE analysis indicated that clMagR-lipase, clMagR-α-AF and clMagR-pullulanase fusion proteins were all successfully expressed in *E*. *coli*. After absorption with Fe_3_O_4_-SiO_2_ nanoparticles, these fusion proteins were effectively purified and showed a single band in SDS-PAGE respectively, which were in accordance with the expected molecular mass (Fig. 4).

The activities of clMagR-lipase, clMagR-α-AF and clMagR-pullulanase fusion proteins were detected. Meanwhile, the activity of free enzymes (without magnetic protein) were purified through Ni-NTA affinity chromatography and tested for comparison. Table 2 presented that the specific activities and purification fold of lipase, α-AF, and pullulanase (purified by Fe_3_O_4_-SiO_2_ nanoparticles) were much higher than that of free enzymes (purified by affinity chromatography), confirming the high specificity of magnetic-based method.

**Table 2:** Enzyme activity of lipase, α-AF and pullulanase. ^a^clMagR-enzymes were purified by iron beads. ^b^clMagR was removed from enzymes. ^c^Free enzymes were purified by Ni-NTA affinity chromatography. ^d^His tag was removed from free enzymes.

Sample	Specific activity (U/mg)	Purification (fold)
Crude extract	187.2 ± 14.5	1.0
clMagR-lipase^a^	1795.3 ± 172.8	9.6
Lipase^b^	1589.1 ± 141.7	8.5
Free lipase^c^	1309.3 ± 112.6	7.0
Free lipase^d^	1487.5 ± 153.8	7.9
Crude extract	15.0 ± 2.2	1.00
clMagR-α-AF^a^	139.5 ± 17.4	9.3
α-AF^b^	118.5 ± 14.3	7.9
Free α-AF^c^	87.0 ± 9.7	5.8
Free α-AF^d^	89.2 ± 8.5	5.9
Crude extract	2.85 ± 0.57	1.00
clMagR-pullulanase^a^	23.09 ± 3.85	8.10
Pullulanase^b^	18.47 ± 3.29	6.48
Free pullulanase^c^	12.20 ± 2.44	4.28
Free pullulanase^d^	12.31 ± 2.23	4.32

The absorption capacity (immobilization efficiency) of iron beads were tested. Different amounts of Fe_3_O_4_-SiO_2_ were added into cell lysates for protein purification. Table 3 illustrated that iron beads had the maximum adsorption capacity of 3.26 mg/g for clMagR-lipase.

**Table 3:** Effect of the amount of iron beads on immobilization efficiency. ^a^clMagR-lipase was used as target protein.

Iron beads (mg)	Adsorbed target protein ^a^ (µg)	Immobilization efficiency (protein/iron beads)
0 mg	0 µg	0 mg/g
0.5 mg	1.43 µg	2.86 mg/g
1 mg	3.26 µg	3.26 mg/g
1.5 mg	3.87 µg	2.58 mg/g
2 mg	4.32 µg	2.16 mg/g

### Separation of the magnetic protein from the target protein

**Figure 5:**
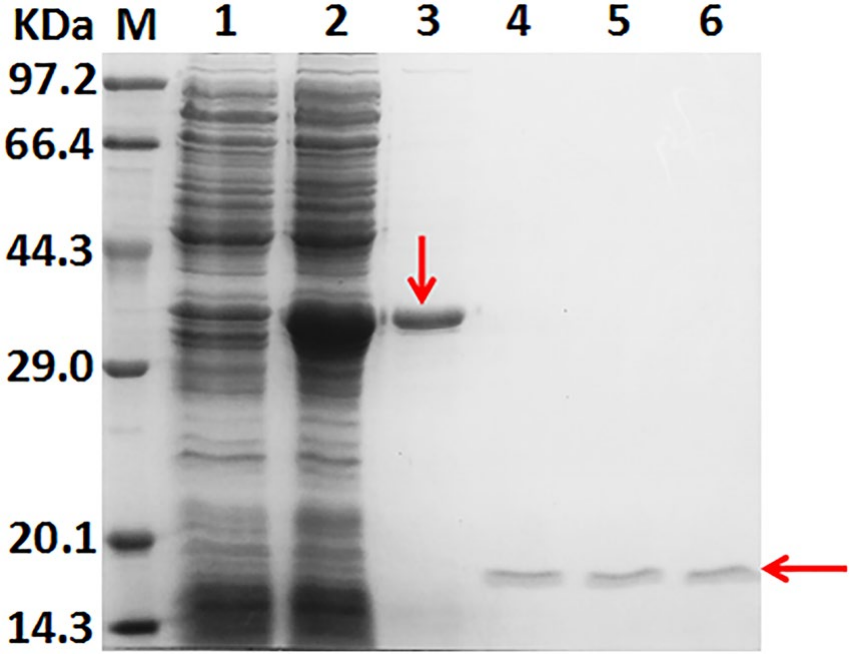
SDS-PAGE analysis of separation of the magnetic protein from the target protein. lane M: standard marker proteins; lane 1:supernatant of cell lysate (BL21-pET28a); lane2: supernatant of cell lysate (BL21-p28aMagR-lipase); lane 3: the purified clMagR-lipase protein (the single band marked with the arrow); lane 4: the purified clMagR-lipase protein was digested with thrombin at 4 °C for 10 h; lane 5: the purified clMagR-lipase protein was digested with thrombin at 20 °C for 3.5 h; lane 6: the purified clMagR-lipase protein was digested with thrombin at 37 °C for 1.5 h (the arrow refers to label-removed lipase (lane 4–6)).

Thrombin was added to cleave the magnetic protein from target protein. As presented in Fig. 5, after digestion at 37 °C for 1.5 h, or 20 °C for 3.5 h, or 4 °C for 10 h, clMagR (absorbed with iron beads) was completely isolated from lipase and removed by centrifugation. The remaining lipase presented a single band on SDS-PAGE with an apparent molecular mass of 20.1 kDa, which was identical to the calculated value. The appropriate cutting temperature can be selected according to the temperature tolerance of the target protein. The specific activity of the remaining lipase was 1589.1 ± 141.7 U/mg, which was higher than that of free lipase (purified by affinity chromatography, without His tag, Table 2). Similarly, the clMagR label was successfully cleaved from α-AF and pullulanase, and the specific activities of α-AF and pullulanase were also higher than activities of their respective free enzymes (purified by affinity chromatography, without His tag, Table 2). Note that the specific activity of clMagR-removed proteins were a little lower than that of clMagR fusion proteins. This may ascribe to enzymatic activity loss during the label-removing process.

### Biochemical characterization of immobilized enzyme

**Figure 6:**
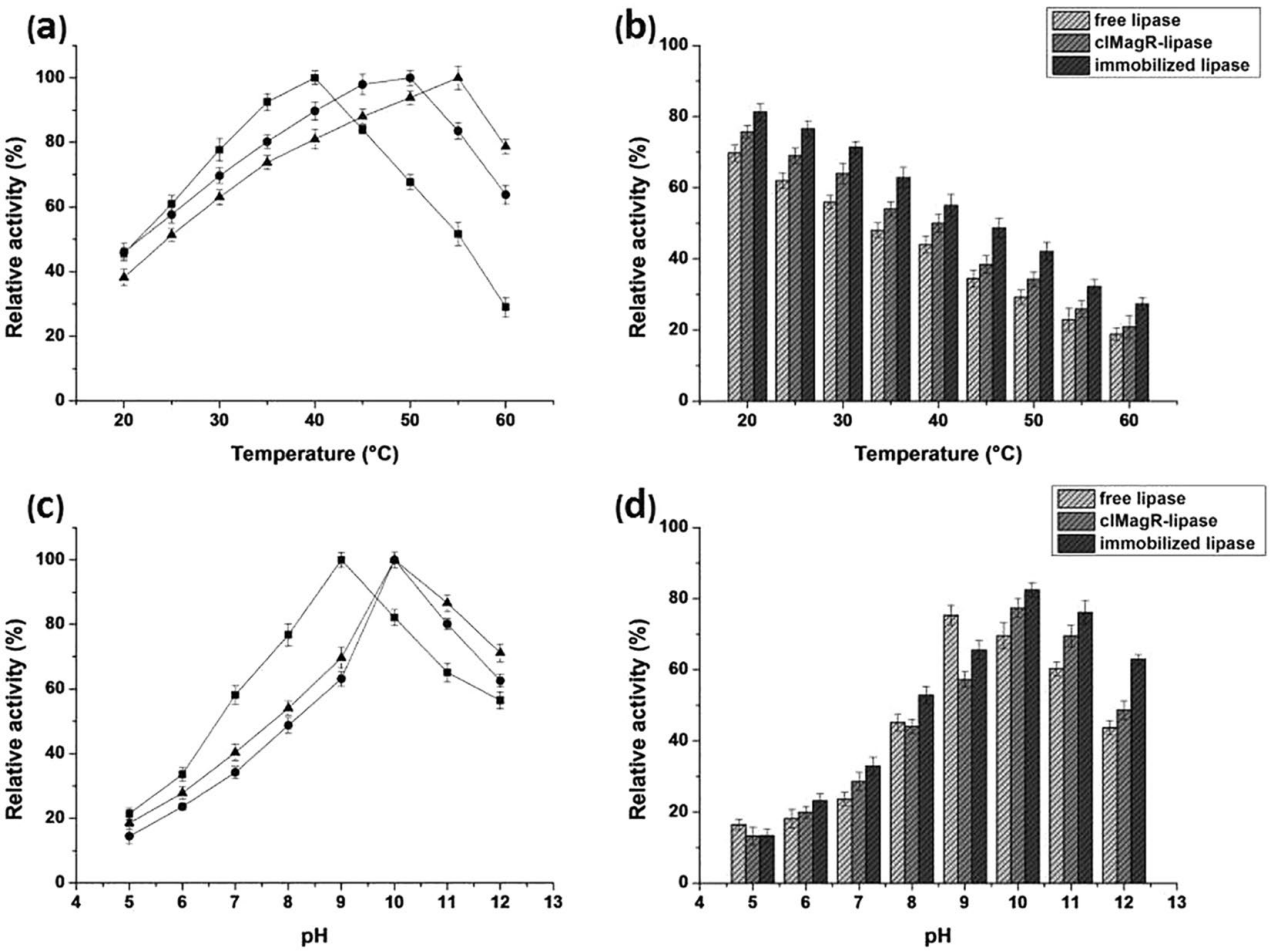
Characterization of free lipase, clMagR-lipase, and immobilized lipase. (**a**) The relative activity of free lipase (■), clMagR-lipase (●), and immobilized lipase (▲) at different temperature; (**b**) Thermostability of the free lipase, clMagR-lipase, and immobilized lipase; (**c**) Relative activity profile of free lipase (■), clMagR-lipase (●), and immobilized lipase (▲) at various pH conditions; (**d**) pH stability of the free lipase, clMagR-lipase, and immobilized lipase.

Upon absorption by iron beads, the clMagR-enzyme could be easily separated by centrifugation and reused for the next batch, thus regarding as immobilized enzyme. The characteristics of free lipase, clMagR-lipase (before purification), and immobilized lipase were investigated. As shown in Fig. 6a, the optimum temperature of the free lipase was 40 °C, but the highest enzymatic activity of clMagR-lipase and immobilized lipase were at 50 °C and 55 °C, respectively. Figure 6c clearly indicates that the free lipase exhibited its maximal activity at pH 9.0, while the maximum activity was at pH 10.0 for clMagR-lipase and immobilized lipase. Thermostability of the free lipase, clMagR-lipase, and immobilized lipase was also studied. All enzymes featured a similar trend: the residual activity decreased with increased temperature. However, the immobilized lipase was obviously more stable than free lipase. At 50 °C, the residual activities of the immobilized lipase, clMagR-lipase, and free lipase were 42%, 34%, and 29%, respectively (Fig. 6b). The pH stability of these lipases were determined by incubating the enzymes in various buffers for 12 h. Figure 6d presents that immobilized lipase displayed much better pH tolerance in alkaline conditions than free enzyme. The magnetic immobilized lipase retained 65% of the optimal activity in excess alkali condition of pH 12.0, while the clMagR-lipase and free lipase maintained only 46% and 42%, respectively.

**Figure 7:**
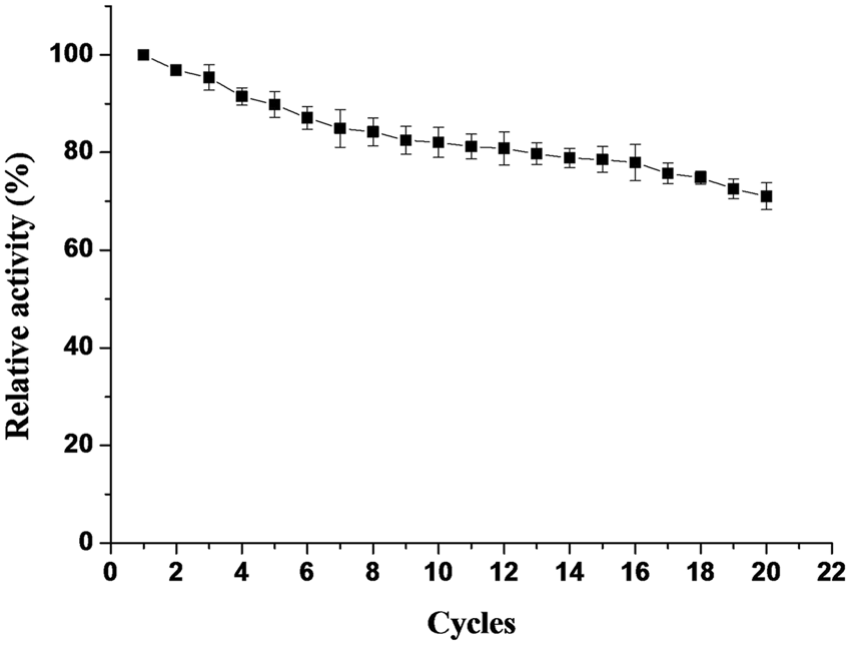
Reusability of the immobilized lipase.

The reusability of clMagR-lipase was studied under constant conditions. As illustrated in Fig. 7, the immobilized lipase maintained nearly 90% of its activity in the first 5 cycles, more than 80% activity after 12 batches, and retained 71% of initial activity even after 20th reuse.

## Discussion

It is well-known that many species can perceive navigation cues from geomagnetic fields for the purpose of guidance or navigation^22^. Recently, Xie *et al*. reported a putative magnetic receptor and a biocompass model, which may form the basis of magnetoreception in animals^4^. This novel magnetic protein is capable of detecting the magnetic field intensity and presents significant orientation preferences to the geomagnetic field. Such a magnetic protein was believed to inspire plenty of potential applications across different fields. In this study, we first used this magnetic protein as a label for purification and immobilization of other proteins.

For practical and economic reasons, integrating protein purification and immobilization steps draws great concerns in biotechnology and industrial applications^23^. Traditional approaches such as immunoaffinity chromatography, metal affinity chromatography, and modified iron oxide nanoparticle immobilization mainly rely on van der Waals interactions, electrostatic interactions, and hydrogen bonding between the protein and the support^24–26^. Therefore, external environment factors, like ionic strength, pH, and temperature have strong impacts on the purification and immobilization process. Here, we established a novel one-step purification and immobilization method based on magnetic absorption. The penetrability of magnetism is independent of external conditions, thus providing a stable separation and immobilization process that can be extensively used in various conditions like extreme environment. Moreover, traditional methods are costly (affinity chromatography column, modification materials), complex to manipulate, time-consuming (sample loading and elution, modification of the support and evaluation), and contaminate the environment (usage of organic solutions, toxicity of metal iron). Magnetic-absorption-based purification and immobilization in this study could potentially overcome all these drawbacks.

In this study, a universal vector was constructed for fusion expression of magnetic protein and target protein, and GFP was employed as reporter protein to validate this purification method. Generally, linker was used to control the distance and prevent interference between two fusion proteins^27,28^. In our work, three linker patterns were designed, and a no-linker pattern was preferred since the low-molecular-size magnetic protein MagR did not affect green fluorescent intensity. Actually, in the subsequent experiments with the target proteins lipase, α-AF, and pullulanase, three linking patterns were all applied. The consistent results (data not shown) indicated that fusion expression with magnetic protein had little effect on target protein activity. Furthermore, to meet high demands of protein application, thrombin site was also introduced, and the target protein could be easily separated from MagR. In addition, the remaining iron beads could be reused continuously and steadily after separating from magnetic protein by boiling in a water bath.

Versatility is of great significance for novel purification approaches. In this work, three enzymes with different molecular sizes were successfully purified and verified the practicability and versatility of this magnetic purification approach. SDS-PAGE analysis suggested that enzymes with lower molecular sizes were easier to purify. The probable reason is that proteins with larger molecular sizes are difficult to pull by MagR. Although the adsorption capacity was not high, using this magnetic-based purification method, the purification fold was much higher than that using Ni-NTA affinity chromatography. Therefore, in the case of low-concentration proteins, the purification method developed in our work has distinct advantages.

The characteristics of immobilized lipase were investigated in this work. In accordance with other immobilization methods^29–31^, the magnetically immobilized lipase exhibited higher thermostability and pH stability. The enhanced thermal stability could be explained by a more rigid conformation, less denaturation and restricted diffusion of lipase aided through immobilization. Similarly, the increased pH stability can be attributed to the interaction between lipase and iron beads (through MagR), which could reduce drastic conformational changes during pH shifting. Reusability is another prominent advantage of immobilized enzymes, which in turn would commercially benefit biocatalyst applications. Many enzymes immobilized on modified iron oxide nanoparticles could be used for no more than 10 cycles^32,33^. This may be attributed to the fact that apart from the detachment between protein and the carrier, the separation of modification material from the support could also result in loss of enzymatic activity. But in the current study, the immobilized lipase maintained over 70% of its initial activity even after 20 cycles.

In summary, the magnetic protein was used in biotechnology for the first time, and a novel one-step protein purification and immobilization method was established in this study. A universal vector and three linker patterns were constructed for fusion expression of magnetic protein and target protein. Several proteins with different molecular sizes were used and verified the practicability of this magnetism-based approach. The detailed report of high purification specificity, improved stability and favorable reusability of immobilized enzyme demonstrated that the magnetic protein-based purification and immobilization method is facile, economical, and stable that can be easily extended to other protein systems. This study is a beneficial attempt and paved a way to apply magnetic protein in biotechnology applications.

## Materials and Methods

### Bacterial strains and plasmids

Two magnetic protein (MagR) genes were used in this study: *clMagR* (GenBank Accession No. XP_005508102.1) and *dMagR* (GenBank Accession No. NP_573062.1), which were synthesized by GenScript (Nanjing, China). *E*. *coli* DH5α (Tiangen, Shanghai, China) was used for construction and conventional amplification of plasmids. *E*. *coli* BL21 (DE3) (Tiangen) was used for protein expression. Plasmid pET-28a(+) (Novagen, Darmstadt, German) was used to construct the protein expression vector. Genes encoding lipase (GenBank Accession No. KF040967.1), α-L-arabinofuranosidase (α-AF, GenBank Accession No. CP002403.1), pullulanase (GenBank Accession No. KJ740392.1) and green fluorescent protein (GFP, GenBank Accession No. KF410615.1) were preserved in our laboratory.

### Chemicals

PrimeSTAR high-fidelity DNA polymerase, T4 DNA ligase, and restriction enzymes were purchased from Takara (Otsu, Japan). ClonExpress II One Step Cloning Kit was provided by Vazyme (Nanjing, China). The substrate 4-nitrophenyl palmitate, substrate 4-nitrophenyl-α-L-arabinofuranoside, substrate pullulan and thrombin were supplied by Sigma-Aldrich (Milwaukee, USA). Other molecular biology reagents and chemicals (analytical grade) were obtained commercially.

### Cloning and expression of clMagR and dMagR in *E*. *coli*

Both *clMagR* and *dMagR* were amplified with the primers clMagRF/clMagRR and dMagRF/dMagRR (Table 4), respectively. After digestion by *Nde* I/*Xho* I and *EcoR* I/*Xho* I, the *clMagR* and *dMagR* genes were cloned into the plasmid pET-28a(+) respectively, which was also digested by the same restriction endonucleases. The positive clones were selected and confirmed by sequencing. The recombinant plasmids pET28a-clMagR and pET28a-dMagR were transformed into *E*. *coli* BL21 (DE3).

**Table 4:** Primers used in this study. ^a^The sequence underlined was introduced.

Designation	Primer sequence (5′-3′)
clMagRF	AGCCATATGATGGCATCTAGCGCATCATC ^a^
clMagRR	GTGCTCGAGTCAGATGTTAAAGCTTTCTC
dMagRF	TCCGAATTCATGGCGACACGTGTGGTGGC
dMagRR	GTGCTCGAGTTACATGCTGAACGATTCGC
MagRF	CGCGGATCCATGGCATCTAGCGCATCATC
MagRR	ACGGAGCTCGATGTTAAAGCTTTCTCCAC
linker1F	ATCGAGCTCAAAGCGAAACTGAAAGAGGA
linker2F	ATCGAGCTCGGCGGAGGTGGCTCTGGCGG
linker1R/linker2R	TTCTCCTTTACTCATATGGCTGCCGCGCGGCACCA
GFP1F/GFP2F	CCATATGAGTAAAGGAGAAGAACT
GFP3F	ATCGAGCTCCTGGTGCCGCGCGGCAGCCATATGAGTAAAGGAGAAGAAC
GFP1R/GFP2R/GFP3R	AGTGCGGCCGCTTATTTGTATAGTTCATCCA
lipaseF	TCCGTCGACCTGGTGCCGCGCGGCAGCCATTCCTCAGGGCATAACCCTG
lipaseR	GTGCTCGAGTTAATTTGTATTTTGTCCGC
AFF	TCCGTCGACCTGGTGCCGCGCGGCAGCCATATGAAAAACTTCAAGATGC
AFR	GTGCTCGAGTCAGTTCAGTGTGATCTCAA
pulluF	TCCGTCGACCTGGTGCCGCGCGGCAGCCATGATTCTACTTCGACTAAAG
pulluR	GTGCTCGAGTTATTGTTTGAGAATAAGCG
28a-lipaseF	CGCGGATCCATGAAACATATAAAAAGCAA
28a-lipaseR	GTGCTCGAGTTAATTTGTATTTTGTCCGC
28a-AFF	CGCGGATCCATGAAAAACTTCAAGATGCT
28a-AFR	GTGCTCGAGTCAGTTCAGTGTGATCTCAA
28a-pulluF	TTCGAGCTCGATTCTACTTCGACTAAAGT
28a-pulluR	GTGCTCGAGTTATTGTTTGAGAATAAGCG

Recombinant cells (BL21-pET28a-clMagR, BL21-pET28a-dMagR) were cultured at 37 °C until the optical density (600 nm) reached 0.6-0.8. IPTG (20 µM) was added to induce clMagR or dMagR expression at 20 °C for 18 h, then the cells were harvested and washed with distilled water. The collected cells were resuspended in TBS buffer (20 mM Tris, 150 mM NaCl, pH 7.5) and disrupted ultrasonically for 15 min. The cell lysate was centrifuged at 4 °C and the supernatant was collected.

### Magnetic validation of clMagR and dMagR

The clMagR and dMagR protein were purified by Fe_3_O_4_-SiO_2_ nanoparticles (BeaverBeads, Suzhou, China). 0.1 mL Fe_3_O_4_-SiO_2_ (10 mg/mL) was added into 0.5 mL cell lysate, followed by incubation for 10 min. The resultant precipitation was harvested by centrifugation, and washed three times with TBS buffer to remove the non-adsorptive proteins. Absorbed proteins were detected by sodium dodecyl sulfate polyacrylamide gel electrophoresis (SDS-PAGE).

### Construction of a universal plasmid

A universal vector was constructed for fusion expression of magnetic protein and target protein. The pET-28a(+) vector was used as the skeleton. Then fragment I containing the whole *clMagR* gene was amplified using primers MagRF/MagRR (Table 4) with *BamH* I and *Sac* I restriction sites respectively. After digestion, fragment I was reclaimed and connected with pET-28a(+), generating the universal vector p28aMagR, which was validated by DNA sequencing.

### Linker design and magnetic purification of GFP

**Figure 8:**
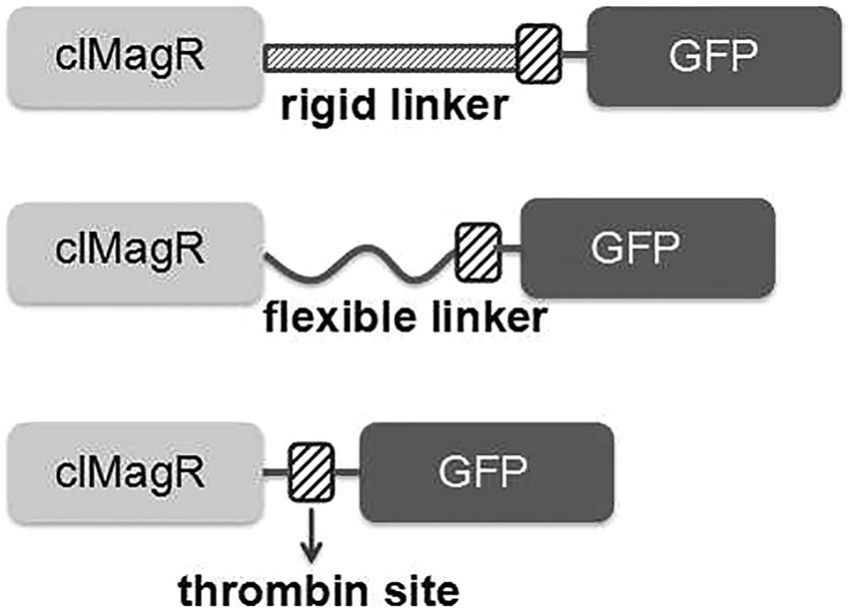
The design patterns of fusion protein.

In order to control the distance and reduce interference between the magnetic protein and target protein, three linker patterns were utilized. Rigid linker (AAAGCGAAACTGAAAGAGGAGGAAGAGC GTAAGCAGCGCGAAGAAGAAGACGTATTAAACGTCTGGAAGAACTGGCG AAACGTAAAGAAGAGGAACGCAAA), flexible linker (GGCGGAGGTGGCTCTGGCGGTGGCGGATCG), and no linker were inserted between magnetic protein and target protein, respectively. Meanwhile, thrombin-cleavage site was added to separate magnetic protein from target protein when necessary. Green fluorescent protein (GFP) was selected as a reporter protein to determine the effect of different linkers on fusion protein activity (Fig. 8).

For plasmid construction, firstly, DNA fragments II and VI including the complete ORF of *gfp* were amplified using primers GFP1F/GFP1R and GFP2F/GFP2R, respectively (Table 4). Secondly, DNA fragments III and VII containing the thrombin digestion sequence + flexible linker and thrombin digestion sequence + rigid linker were synthesized, and applied as templates to amplify DNA fragment IV (primers linker1F/linker1R) and VIII (linker2F/linker2R), respectively. Thirdly, using fragments II + IV, and VI + VIII as templates, fragments V and IX were obtained with primers linker1F/GFP1R and linker2F/GFP2R, respectively. Next, the vector p28aMagR was digested by *Sac* I and *Not* I, and then integrated with the uniformly treated fragments V and IX, generating vectors p28aMagR-flexible linker-GFP and p28aMagR-rigid linker-GFP, respectively. Similarly, the *gfp* gene was amplified with the primers GFP3F/GFP3R. After digestion by *Sac* I/*Not* I, the *gfp* gene was cloned into the plasmid p28aMagR to obtain vector p28aMagR-GFP.

The recombinant plasmids p28aMagR-flexible linker-GFP, p28aMagR-rigid linker-GFP, and p28aMagR-GFP were transformed into *E*. *coli* BL21 (DE3) respectively. The vector p28aMagR was expressed in *E*. *coli* BL21 (DE3) as control. Transformants were grown and induced with 20 µM IPTG at 20 °C for 10 h. Cells were harvested, washed two times with TBS buffer, and disrupted by ultrasonication. The fusion protein was purified by Fe_3_O_4_-SiO_2_ nanoparticles as described above. Subsequently, the crude extract and the purified fusion protein were analyzed by SDS-PAGE. Meanwhile, 200 μL of MagR-GFP, MagR-flexible linker-GFP, and MagR-rigid linker-GFP solution was transferred to a 96-well microplate, respectively, and then placed in a BioTek^TM^ Cytation^TM^ 3 Cell Imaging Multi-Mode Reader (Vermont, USA) with 485 nm excitation and 520 nm emission. GFP signals were read before being processed in Microsoft Excel^34^.

### Purification and activity detection of lipase, α-AF and pullulanase

In order to confirm the universality of this purification method, lipase, α-AF, and pullulanase with different molecular sizes were selected as target proteins. Primers lipaseF/lipaseR, AFF/AFR, pulluF/pulluR (Table 4), with *Sal* I and *Xho* I digestion sites, were synthesized to amplify the complete ORF of genes coding lipase, α-AF and pullulanase, respectively. Meanwhile, a thrombin digestion sequence was introduced in the 5’ end of these genes. The purified PCR products were digested with *Sal* I and *Not* I and integrated into universal vector p28aMagR, creating p28aMagR-lipase, p28aMagR-α-AF and p28aMagR-pullulanase, respectively.

Meanwhile, the coding sequence of lipase, α-AF and pullulanase were amplified with the primers 28a-lipaseF/28a-lipaseR, 28a-AFF/28a-AFR, and 28a-pulluF/28a-pulluR, respectively. These PCR products were digested and cloned into plasmid pET28a (+), creating pet28a-lipase, pet28a-α-AF, and pet28a-pullulanase as comparison.

**Figure 9:**
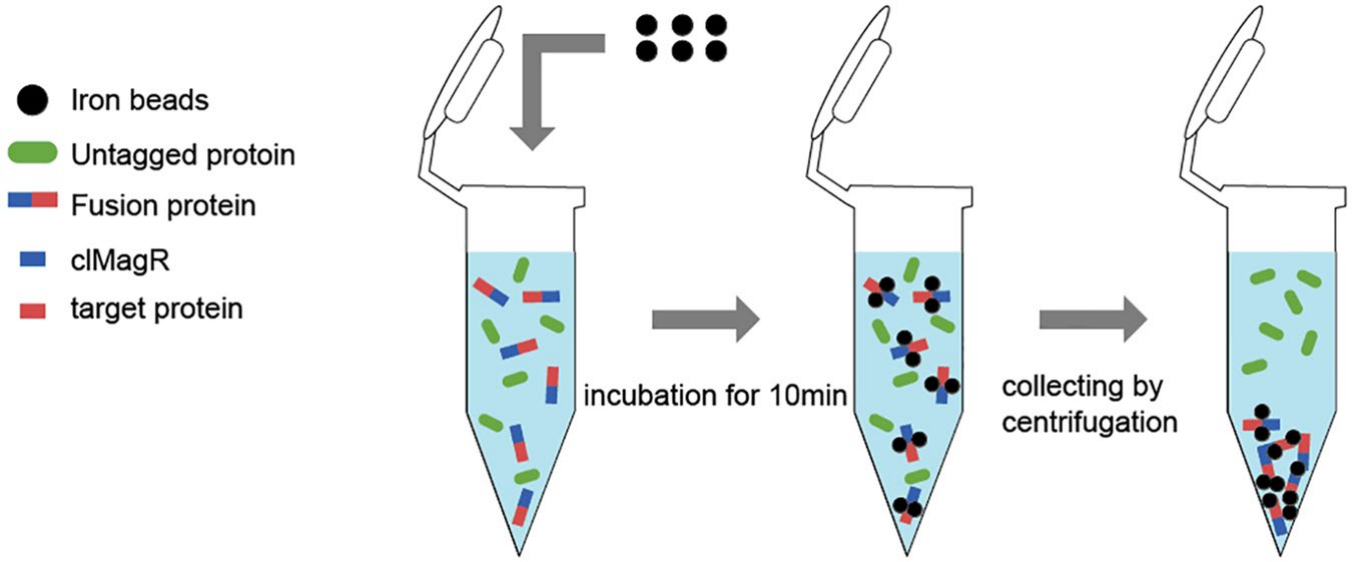
The purification flow chart of target protein of fusion magnetic protein.

The recombinant vectors were transformed into *E*. *coli* BL21 (DE3) respectively. IPTG (0.1 mmol/L) was added to induce the cultures at 20 °C for 16 h. The cells were collected by centrifugation, washed with TBS buffer twice, and then disrupted by ultrasonication. The fusion proteins (MagR + lipase/α-AF/pullulanase) were purified by Fe_3_O_4_-SiO_2_ nanoparticles as described above (Fig. 9). Subsequently, the crude extracts and the purified fusion proteins were analyzed by SDS-PAGE. The free enzymes (without MagR) were purified using Ni-NTA sepharose column (Qiagen, Dusseldorf, Germany) according to the manufacturer’s protocol^35^. The protein concentrations were determined using BCA Protein Assay kit (Biotech Well, Shanghai, China) with bovine serum albumin (BSA) as a standard.

Lipase activity was determined according to the method described by Cai *et al*.^36^. One unit of lipase activity is defined as the amount of enzyme liberate 1 μmol p-nitrophenol per min from 4-nitrophenyl palmitate under the assay conditions. The α-AF activity was assayed in accordance with Yang *et al*.^37^. One unit of α-AF activity is defined as the amount of enzyme releasing 1 μmol p-nitrophenol per min using 4-nitrophenyl α-L-arabinofuranoside as substrate. The activity of pullulanase was estimated using the 3,5-dinitrosalicylic acid (DNS) method^38^. One unit of pullulanase activity is defined as the amount of enzyme required to release 1 µmol of glucose equivalent of reducing sugar per minute under the assay conditions.

To study the absorption capacity (immobilization efficiency) of iron beads, 0 mg, 0.5 mg, 1 mg, 1.5 mg and 2 mg Fe_3_O_4_-SiO_2_ nanoparticles were added into the 1 mL cell lysate respectively, and incubated at room temperature for 10 min. The resulting precipitate was harvested and washed three times with TBS buffer to remove the non-adsorptive proteins. The bead-adsorbed protein was boiled for 5 min, and separated from iron beads by centrifugation. The supernatant protein concentrations were determined using BCA Protein Assay kit (Biotech Well, Shanghai, China) with bovine serum albumin (BSA) as a standard.

### Separation of magnetic protein from target proteins

*E*. *coli* BL21 expressing p28aMagR-lipase, p28aMagR-α-AF, or p28aMagR-pullulanase were harvested and disrupted ultrasonically. The fusion proteins were purified with iron beads and then resuspended in 20 mM Tris-HCl buffer (NaCl 150 mM, pH 8.0). Subsequently, appropriate amount of thrombin (about 0.2 U) was added into 0.1 mL of fusion protein solution. The reaction mixture was incubated at different temperatures (4 °C, 20 °C, or 37 °C) for a certain period of time (10 h, 3.5 h or 1.5 h). Finally, the iron beads with MagR protein were removed by centrifugation. The supernatants containing lipase, α-AF, or pullulanse were analyzed and detected by activity measurement respectively. Meanwhile, the free enzymes (with His tag, without MagR) were purified using Ni-NTA sepharose column. Subsequently, appropriate amount of thrombin (about 0.5 U) was added into 0.1 mL of free enzymes solution. The reaction mixture was incubated at 37 °C for 1.5 h. Finally, the activities of free enzymes (without His tag) were detected for comparison.

### One-step purification and immobilization of lipase

The purification and immobilization of lipase was realized by the physical adsorption of iron beads and magnetic protein. The enzymatic properties and reusability of fusion lipase (clMagR-lipase) that immobilized on iron beads were analyzed. The characteristics of free lipase (purified by Ni-NTA affinity chromatography) and clMagR-lipase (before purification) were investigated for comparison. The optimum temperature for the immobilized lipase and free lipase was determined under standard assay conditions at different temperature (20–60 °C) (pH 9.0). The thermostability was evaluated by measuring the residual activity after incubating the enzymes at various temperatures ranging from 20 °C to 60 °C for 12 h in Tris-HCl buffer (pH 9.0). Similarly, to determine the optimum pH, immobilized lipase and free lipase activities were studied over a pH range of 5.0–12.0 at 40 °C. The pH stability was investigated by incubating the enzyme in different buffers for 12 h at 4 °C and the residual activity was measured as previously described. The following buffers were used: 50 mM citrate buffer (pH 5.0–6.0), 50 mM sodium phosphate buffer (pH 6.0–7.0), 50 mM Tris–HCl buffer (pH 8.0–9.0), and 50 mM glycine-NaOH buffer (pH 10.0–12.0).

The reusability of the immobilized lipase was measured under constant conditions. Immobilized enzyme was recovered from the reaction media by centrifugal separation and washed with TBS buffer for the next batch. The reaction was conducted for a total of 20 batches, where the activity of the first run was defined as 100%.
